# Cancer and Chemotherapy Contribute to Muscle Loss by Activating Common Signaling Pathways

**DOI:** 10.3389/fphys.2016.00472

**Published:** 2016-10-19

**Authors:** Rafael Barreto, Giorgia Mandili, Frank A. Witzmann, Francesco Novelli, Teresa A. Zimmers, Andrea Bonetto

**Affiliations:** ^1^Department of Surgery, Indiana University School of MedicineIndianapolis, IN, USA; ^2^Centre for Experimental and Clinical Studies, University of TorinoTorino, Italy; ^3^Department of Molecular Biotechnology and Health Sciences, University of TorinoTorino, Italy; ^4^Department of Cellular and Integrative Physiology, Indiana University School of MedicineIndianapolis, IN, USA; ^5^Simon Cancer Center, Indiana University School of MedicineIndianapolis, IN, USA; ^6^Center for Cachexia Research Innovation and Therapy, Indiana University - Purdue University IndianapolisIndianapolis, IN, USA; ^7^Department of Otolaryngology, Head and Neck Surgery, Indiana University School of MedicineIndianapolis, IN, USA

**Keywords:** Folfiri, C26, proteomics, muscle, inflammation, cachexia, mitochondria, mitochondrial fusion and fission

## Abstract

Cachexia represents one of the primary complications of colorectal cancer due to its effects on depletion of muscle and fat. Evidence suggests that chemotherapeutic regimens, such as Folfiri, contribute to cachexia-related symptoms. The purpose of the present study was to investigate the cachexia signature in different conditions associated with severe muscle wasting, namely Colon-26 (C26) and Folfiri-associated cachexia. Using a quantitative LC-MS/MS approach, we identified significant changes in 386 proteins in the quadriceps muscle of Folfiri-treated mice, and 269 proteins differentially expressed in the C26 hosts (*p* < 0.05; −1.5 ≥ fold change ≥ +1.5). Comparative analysis isolated 240 proteins that were modulated in common, with a large majority (218) that were down-regulated in both experimental settings. Interestingly, metabolic (47.08%) and structural (21.25%) proteins were the most represented. Pathway analysis revealed mitochondrial dysfunctions in both experimental conditions, also consistent with reduced expression of mediators of mitochondrial fusion (OPA-1, mitofusin-2), fission (DRP-1) and biogenesis (Cytochrome C, PGC-1α). Alterations of oxidative phosphorylation within the TCA cycle, fatty acid metabolism, and Ca^2+^ signaling were also detected. Overall, the proteomic signature in the presence of both chemotherapy and cancer suggests the activation of mechanisms associated with movement disorders, necrosis, muscle cell death, muscle weakness and muscle damage. Conversely, this is consistent with the inhibition of pathways that regulate nucleotide and fatty acid metabolism, synthesis of ATP, muscle and heart function, as well as ROS scavenging. Interestingly, strong up-regulation of pro-inflammatory acute-phase proteins and a more coordinated modulation of mitochondrial and lipidic metabolisms were observed in the muscle of the C26 hosts that were different from the Folfiri-treated animals. In conclusion, our results suggest that both cancer and chemotherapy contribute to muscle loss by activating common signaling pathways. These data support the undertaking of combination strategies that aim to both counteract tumor growth and reduce chemotherapy side effects.

## Introduction

According to the American Cancer Society, colorectal cancer represents the third leading cause of cancer-related deaths in the United States (American Cancer Society, 2015). Each year, about 150,000 Americans are diagnosed with colorectal cancer, and one third of those individuals die from the disease (Siegel et al., 2015). Colorectal cancer therapy frequently includes treatment with 5-fluorouracil (5-FU), Leucovorin (LV) and CPT-11, a combination also known as Folfiri. Among the several side effects frequently associated with the administration of Folfiri, increased fatigue represents one of the most common (Montagnani et al., 2011). Notably, cachexia poses a serious problem for patients' quality of life and survival.

Cachexia is a devastating condition associated with several types of malignant cancers and is comorbid in 22–55% of all colorectal cancer cases (Thoresen et al., 2013). A major contributor of colorectal cancer morbidity and mortality, cachexia is primarily responsible for body and muscle weight loss and correlates with tumor burden, increased pro-inflammatory cytokine levels, fatigue, and reduced response to chemo- and radio-therapy (Ravasco et al., 2007; Bapuji and Sawatzky, 2010; Fearon et al., 2012). Studies suggest that cachexia may result from tumor-host interactions or activation of an inflammatory response. We reported that blocking muscle wasting can prolong life even in the absence of effects on tumor growth (Benny Klimek et al., 2010). Hence, targeting cachexia *per se* could improve outcomes and enhance tumor-free survival.

Notably, although the molecular mechanisms responsible for the development of cancer cachexia have been studied for quite some time, it is not completely clear whether cancer treatments also play a causative role in the development of cachexia. Along this line, the use of cytotoxic and anti-proliferative drugs for the treatment of cancer is frequently accompanied by several pronounced side effects, including nausea, diarrhea, anorexia and fatigue, all of which are responsible for significantly decreasing the quality of life of cancer patients and increasing morbidity and mortality. Interestingly, findings show that chemotherapy can promote cachexia development regardless of its effects on tumor growth (Damrauer et al., 2008; Garcia et al., 2008).

Furthermore, it has also been reported that cancer patients affected with muscle depletion (regardless of body weight) are more susceptible to developing severe drug-associated toxicity and show a poorer prognosis. Conversely, subjects with higher muscle mass or not showing sarcopenia are generally more resistant and may tolerate higher doses of chemotherapy (Antoun et al., 2010; Prado et al., 2011; Thoresen et al., 2013; Jung et al., 2015; Stene et al., 2015). We recently reported that Folfox and Folfiri, which are chemotherapeutics utilized for the treatment of solid tumors, may contribute to the development of cachexia and muscle weakness by promoting oxidative stress-associated MAPK activation and by affecting the muscle mitochondrial pool (Barreto et al., 2016). Despite this, it is still partially unknown whether chemotherapy directly promotes cachexia and whether this occurs by activating the same molecular mechanisms associated with muscle wasting in the presence of a tumor.

The purpose of this study was to identify and compare signaling pathways associated with cancer- and chemotherapy-induced muscle wasting based on differences in protein expression. We performed LC-MS/MS-based proteomic profiling of quadriceps muscle from mice bearing the Colon-26 (C26) adenocarcinoma and from mice administered Folfiri for 5 weeks (Bonetto et al., 2012; Barreto et al., 2016). We then employed a software-based analysis to identify upstream regulators and causal networks associated with known diseases and functions. Together, this study represents one of the first attempts to perform a proteomic-based investigative approach in the skeletal muscle of mice that are affected with cachexia with potential translational implications for tumor-derived cachexia, as well as muscle depletion due to chemotherapy.

## Materials and methods

### Animals

All experiments were conducted with the approval of the Institutional Animal Care and Use Committee at Indiana University School of Medicine and were in compliance with the National Institutes of Health Guidelines for Use and care of Laboratory Animals. In order to investigate the effect of chemotherapy on muscle mass, 8-week old CD2F1 male mice (*n* = 8; Harlan, Indianapolis, IN) were administered Folfiri, a combination of 5-fluorouracil (50 mg/kg), leucovorin (90 mg/kg) and CPT-11 (24 mg/kg), intraperitoneally (i.p.), twice a week for five consecutive weeks (Barreto et al., 2016). Based on previous findings, the amounts of drugs that were delivered to the experimental animals were not exceeding clinically relevant concentrations (Barreto et al., 2016). Control mice received an equal volume of vehicle. All drugs were purchased from Sigma Aldrich (St. Louis, MO). For the cancer cachexia model, Colon26 cells were cultured in DMEM medium supplied with 10% fetal bovine serum and 1% penicillin/streptomycin and maintained in a 5% CO_2_, 37°C humidified incubator. Cells were passaged when sub-confluent, and 1 × 10^6^ cells were injected subcutaneously in 8-week old CD2F1 male mice (*n* = 8). Non-tumor bearing normal mice were used as controls. Mice were weighed daily then euthanized under light isoflurane anesthesia. Tissues were collected and weighed, then snap frozen in liquid nitrogen and stored at −80°C for further studies.

### Sample preparation

DL-Dithiothreitol (DTT), urea, triethylphosphine, iodoethanol, and ammonium bicarbonate (NH_4_HCO_3_) were purchased from Sigma-Aldrich (St. Louis, MO, USA). LC-MS grade 0.1% formic acid in acetonitrile (ACN) and 0.1% formic acid in water (H_2_O) were purchased from Burdick and Jackson (Muskegon, MI, USA). Modified sequencing grade porcine trypsin was obtained from Princeton Separations (Freehold, NJ, USA). To 70 mg of each of the liquid N_2_-pulverized quadriceps muscle samples, 400 μL of 8 M urea/10 mM DTT was added. Each tissue sample was treated by light sonication and mixed for 1 h at room temperature, followed by centrifugation at 13,000 rpm for 10 min. The protein concentration was measured by Bradford assay (Bradford, 1976). 20 μL was removed and 20 μL of 100 mM ammonium carbonate, pH 10.8, was added to the samples. 40 μL of reduction/alkylation cocktail (97.5% acetonitrile, 2% iodoethanol, and 0.5% triethylphosphine) was added to each sample, and samples were incubated in a 37°C incubator for 1.5 h. The samples were speed vacuumed to dryness overnight, and the dry pellets were resuspended in 50 μL ammonium bicarbonate. 2.5 μg trypsin in 100 μL ammonium bicarbonate was added to each sample, and they were incubated at 37°C for 4 h. 2.5 μg trypsin in 50 μL ammonium bicarbonate was then added to each sample, and they were incubated at 37°C overnight.

### LC-MS/MS

The digested samples were analyzed using a Thermo Scientific Orbitrap Velos Pro mass spectrometer coupled with a Surveyor autosampler and MS HPLC system (Thermo Scientific). Tryptic peptides were injected onto a C18 reversed phase column (TSKgel ODS-100V, 3 μm, 1.0 × 150 mm) at a flow rate of 50 μL/min. The mobile phases A and B were LC-MS grade H_2_O with 0.1% formic acid and ACN with 0.1% formic acid, respectively. The gradient elution profile was as follows: 5% B for 5 min, 10–35% B for 150 min, 35–80% B for 10 min, 80% B for 10 min, and 5% B for 5 min. The data were collected in the “Data dependent MS/MS” mode of FT-IT with the ESI interface using normalized collision energy of 35% (CID). Dynamic exclusion settings were set to repeat count 1, repeat duration 30 s, exclusion duration 120 s, and exclusion mass width 10 ppm (low) and 10 ppm (high).

### Peptide/protein identification and quantification

The acquired data were searched against the UniProt protein sequence database of MOUSE (released on 06/24/2015) using X1 Tandem algorithms in the Trans-Proteomic Pipeline (TPP, v. 4.6.3) (http://tools.proteomecenter.org/software.php). General parameters were: parent monoisotopic mass error set as 10 ppm, cleavage semi set as yes, missed cleavage sites set at 2, and static modification set as + 44.026215 Da on Cysteine. The searched peptides and proteins were validated by PeptideProphet (Ma et al., 2012) and ProteinProphet (Nesvizhskii et al., 2003) in the TPP. Only proteins and peptides with protein probability ≥0.9000 and peptide probability ≥0.8000 were reported. False discovery rate (FDR) was estimated by a non-parametric concatenated randomized target-decoy database search (Elias and Gygi, 2007). For this experiment and those TPP settings, protein identification FDR was < 0.2%. Protein quantity was determined using an in-house label-free quantification software package, IdentiQuant^XL^, developed to individually and accurately align the retention time of each peptide and to apply multiple filters for exclusion of unqualified peptides to enhance label-free protein quantification. As previously described in detail (Lai et al., 2011), peptide retention time determination using clustering, extraction of peptide intensity using MASIC (Monroe et al., 2008), peptide coefficient of variation calculation, and peptides correlation were all conducted within the software platform to “filter out” unqualified peptides. Using only qualified peptides, protein intensity was calculated using the formula: Protein Intensity = (intensity of peptide 1)/(peptide 1 sharing times) + (intensity of peptide n)/(peptide n sharing times). For a peptide shared by different proteins, the intensity of this peptide was divided by the number of times the peptide was shared. Raw data were deposited in the PeptideAtlas database and are available through identifier PASS00863 (http://www.peptideatlas.org/PASS/PASS00863).

### Western blotting

Total protein extracts were obtained by homogenizing 100 mg quadriceps muscle tissue in RIPA buffer (150 mM NaCl, 1.0% NP-40, 0.5% sodium deoxycholate, 0.1% SDS, and 50 mM Tris, pH 8.0) completed with protease (Roche, Indianapolis, IN) and phosphatase (Thermo Scientific, Rockford, IL) inhibitor cocktails. Cell debris were removed by centrifugation (15 min, 14000 g) and the supernatant collected and stored at −80°C. Protein concentration was determined using the BCA protein assay method (Thermo Scientific, Rockford, IL). Protein extracts (30 μg) were then electrophoresed in 4–15% gradient SDS Criterion TGX precast gels (Bio-Rad, Hercules, CA). Gels were transferred to nitrocellulose membranes (Bio-Rad, Hercules, CA). Membranes were blocked with SEA BLOCK blocking reagent (Thermo Scientific, Rockford, IL) at room temperature for 1 h, followed by an overnight incubation with diluted antibody in SEA BLOCK buffer containing 0.2% Tween-20 at 4°C with gentle shaking. After washing with PBS containing 0.2% Tween-20 (PBST), the membrane was incubated at room temperature for 1 h with either Anti-rabbit IgG (H+L) DyLight 800 or Anti-mouse IgG (H+L) DyLight 600 (Cell Signaling Technologies, Danvers, MA). Blots were then visualized with Odyssey Infrared Imaging System (LI-COR Biosciences, Lincoln, NE). Optical density measurements were taken using the Gel-Pro Analyzer software. Antibodies used were OPA-1 (#80471), Mitofusin-2 (#9482), DRP-1 (#8570), Cytochrome C (#11940) from Cell Signaling Technologies (Danvers, MA), PGC-1α (#ab3242) from Abcam (Cambridge, MA) and α-Tubulin (#12G10) from Developmental Studies Hybridoma Bank (Iowa City, IA).

### Statistics

Comparisons among tissue weights reported in Table 1 were carried out using Student's *t*-test. A value of *p* ≤ 0.05 was considered statistically significant. As for the LC-MS/MS proteomic study, only the proteins identified with at least two peptides and with −1.5 ≥ fold change (FC) ≥ +1.5 were included in the analysis. Comparative analysis between the two datasets was carried over by means of Correlation Engine (Illumina, San Diego, CA). Finally, statistically significant and differentially expressed proteins (FDR < 5%) from both datasets were imported into Ingenuity Pathway Analysis (IPA; Qiagen, Valencia, CA) to identify significant pathways, upstream regulators and causal networks associated with known diseases and functions.

**Table 1:** **Body and tissue weights in tumor-bearing mice and chemotherapy-treated animals**.

	**Cancer**		**Chemotherapy**	
	**Control (*n* = 8)**	**C26 (*n* = 8)**	**Vehicle (*n* = 8)**	**Folfiri (*n* = 8)**
IBW	25.35 ± 1.61	26.60 ± 1.12	26.70 ± 1.70	24.6 ± 2.70
FBW	25.62 ± 1.80	22.4 ± 2.63^a^	28.80 ± 1.70	24.6 ± 3.10^bb^
GSN	0.57 ± 0.02	0.44 ± 0.03^aaa^	0.55 ± 0.04	0.49 ± 0.01^bb^
Quadriceps	0.74 ± 0.05	0.56 ± 0.08^aaa^	0.77 ± 0.05	0.62 ± 0.02^bbb^
Heart	0.53 ± 0.02	0.45 ± 0.06^aa^	0.57 ± 0.04	0.61 ± 0.02
Liver	4.52 ± 0.23	4.65 ± 0.64	4.88 ± 0.52	4.95 ± 0.34
Spleen	0.28 ± 0.03	1.09 ± 0.81^aaa^	0.27 ± 0.03	0.81 ± 0.12^bbb^
Fat	2.25 ± 0.35	0.89 ± 0.45^aaa^	2.89 ± 0.54	1.08 ± 0.32^bbb^

*Data are expressed as means ± SD. Initial body weight (IBW) and Final body weight (FBW) are reported in grams (g). Gastrocnemius (GSN), quadriceps, liver, spleen and fat are reported as weight/100 mg IBW. Significance of the differences*:

a *p < 0.05*,

aa *p < 0.01*,

aaa *p < 0.001 vs. Control*;

bb *p < 0.01*,

bbb *p < 0.001 vs. Vehicle*.

## Results

### Tumor growth and chemotherapy administration promote the occurrence of severe cachexia

In order to limit the variability across the different animal models, both tumor hosts and animals treated with chemotherapy were sacrificed when muscle loss was comparable (about 15%) and resembling a condition of severe cachexia, as previously shown (Bonetto et al., 2011). In particular, CD2F1 male mice (*n* = 8) were injected s.c. with C26 adenocarcinoma cells and weighed daily. After 14 days from tumor injection, tumor hosts were sacrificed when their final body weight reached about 87% of the control animals (*p* < 0.01) (Table 1) (Bonetto et al., 2011). In this setting, marked muscle wasting was observed, both at the gastrocnemius and quadriceps level (−23 and −25% vs. control, respectively; *p* < 0.001). A condition associated with cardiac atrophy was also displayed and is associated with tumor growth (−15% vs. control; *p* < 0.01). Similar to that previously described in the same experimental model of cancer cachexia (Bonetto et al., 2011, 2012), severe depletion of adipose tissue (−61% vs. control; *p* < 0.001), as well as splenomegaly (+289% vs. control; *p* < 0.001), were observed (Table 1). In separate set of experiments, CD2F1 normal mice were administered Folfiri (twice/week) for 5 weeks, and body weight was monitored daily (Barreto et al., 2016). At the time of sacrifice, the chemotherapy-treated mice showed significant loss of body weight (−15%, *p* < 0.01), consistent with depletion of gastrocnemius (−11%, *p* < 0.01), quadriceps (−20%, *p* < 0.001), and fat (−63%, *p* < 0.001) mass. Interestingly, the heart mass was not affected by chemotherapy administration. Similar to the tumor-bearing animals, a dramatic increase in spleen size (+200% vs. vehicle, *p* < 0.001) was also observed (Table 1).

### C26 tumor and folfiri influence the skeletal muscle proteome

In order to elucidate the mechanisms responsible for muscle wasting in cancer-associated cachexia and chemotherapy-induced cachexia, we investigated the muscle proteome in C26 hosts and in mice treated with Folfiri. By taking advantage of a LC-MS/MS quantitative approach, we detected 422 proteins in the muscle of animals carrying the C26 tumor and 511 proteins in the muscle of mice exposed to chemotherapy (Tables S1–S3). Of note, 269 proteins, among the ones identified with at least two peptides and with −1.5 ≥ fold change (FC) ≥ +1.5, were differently expressed in the C26 hosts, while 386 were significantly (*p* < 0.05) modulated in the muscle of animals treated with Folfiri (Table S4).

**Figure 1:**
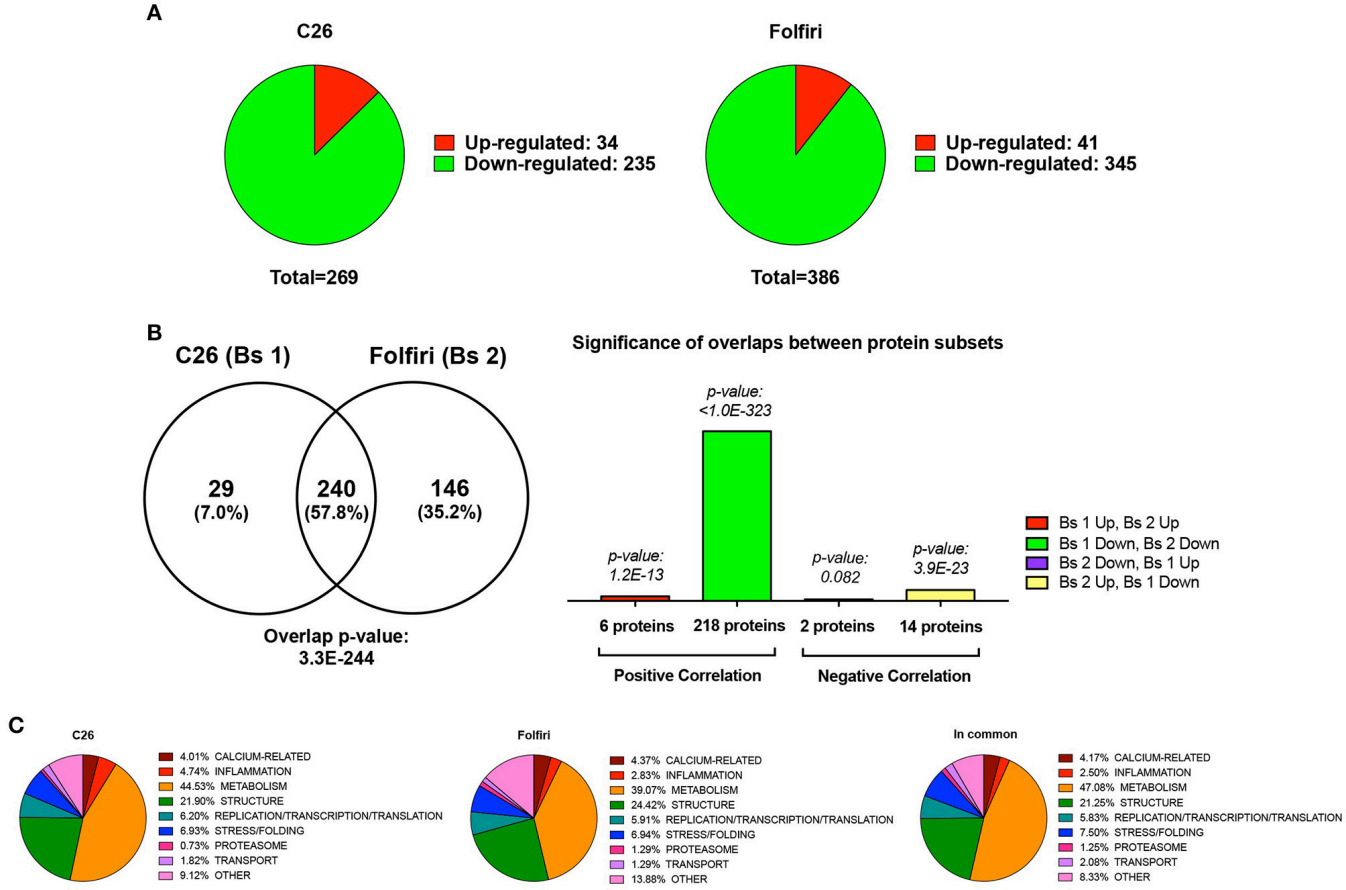
**Cancer and chemotherapy promote the down-regulation of 235 and 345 muscle proteins, respectively**. **(A)** Pie charts showing the number of proteins that are down-regulated (green) or up-regulated (red) following tumor growth (C26, left) or chemotherapy (Folfiri, right). **(B)** Comparative analysis between C26 and Folfiri biosets (Bs 1 and Bs 2, respectively). The Venn diagram (left) shows the number of proteins that are modulated in common or in the presence of either C26 or Folfiri. The overlap *p*-value, indicating the statistically significant overlap between the two datasets, is also reported. Analogously, the significance of the overlap between different protein subsets within the group of proteins modulated in both subsets is also presented (right). **(C)** The proteins detected in the C26 (left) and Folfiri (middle) datasets, or modulated in common (right) were classified based on their function and/or pathway and distributed as shown in the pie charts. The percentage is expressed over the total number in proteins in each dataset.

In particular, among the 269 proteins modulated in the cancer setting, 235 were down-regulated, while 34 were up-regulated (Figure 1A, Table S4). Analogously, following chemotherapy administration, a large majority of proteins (345) were down-regulated, while only a small subset of proteins (41) was up-regulated or expressed exclusively in the muscle of animals receiving Folfiri (Figure 1A, Table S4). Comparative analysis performed by means of Illumina Correlation Engine identified 240 proteins that were modulated in both experimental conditions (*p* = 3.3E-244), with a significant positive correlation (218 proteins; *p* < 1.0E-323) for the proteins that were down-regulated in both experimental models (Figure 1B). Quite interestingly, members of metabolic pathways (39.1% in Folfiri, 44.5% in C26) and structural proteins (24.4% in Folfiri, 21.9% in C26) were the most represented in both subsets (Figure 1C, left and middle panels). A similar situation was also observed among the 240 proteins modulated in common, with metabolic and structural proteins representing the large majority and totaling about 68% (Figure 1C, right).

### Muscle mitochondrial dysfunctions are the main event associated with tumor growth or chemotherapy treatment

**Figure 2:**
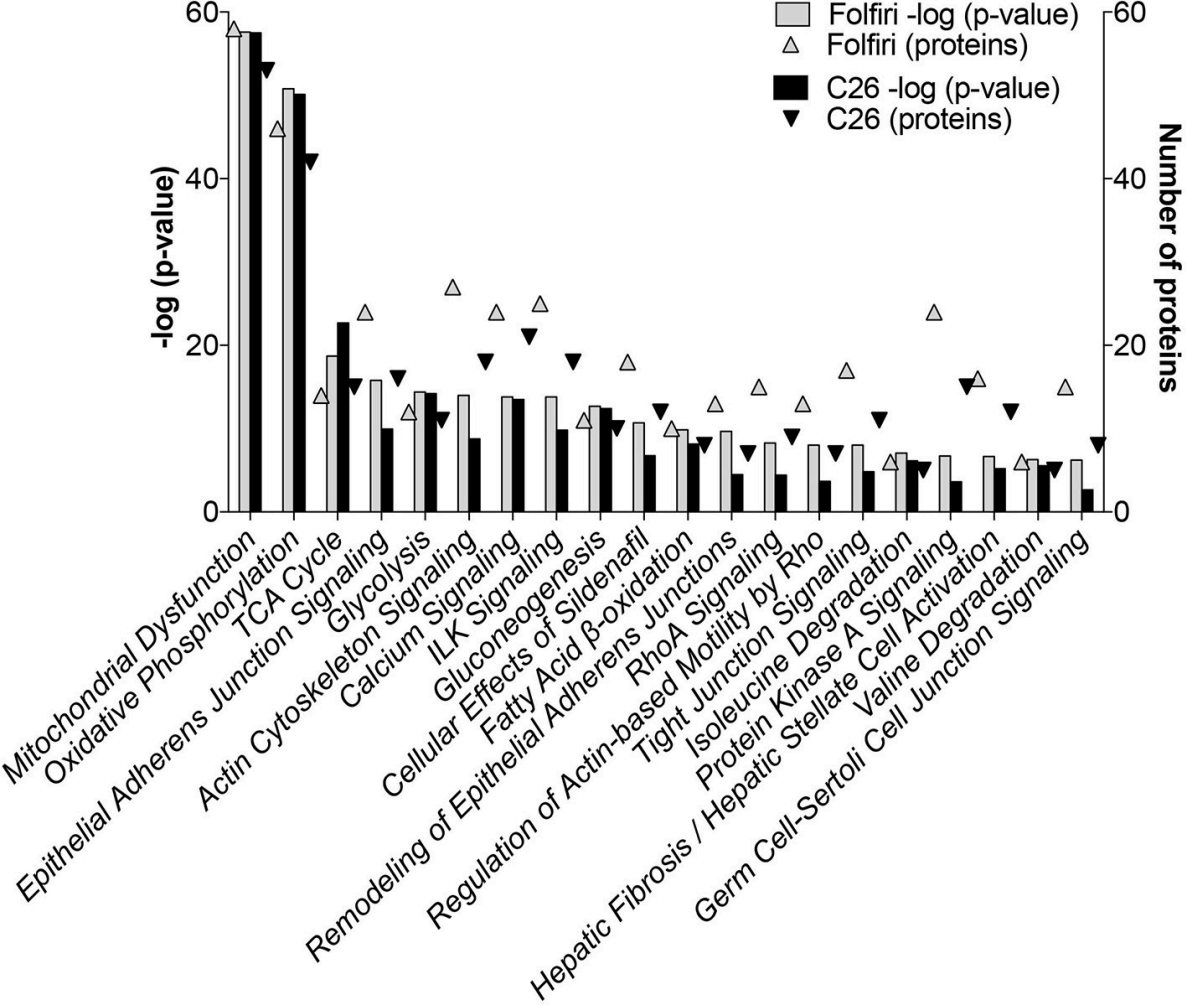
**Pathway analysis of muscle proteomic profiling in cancer or chemotherapy-induced cachexia**. By utilizing the IPA software, the C26 and Folfiri datasets were subjected to pathway analysis. The pathways were ranked based on their overlap *p*-value (bars). Top-20 pathways are reported in the diagram, along with the number of proteins modulated within each pathway (triangles).

**Figure 3:**
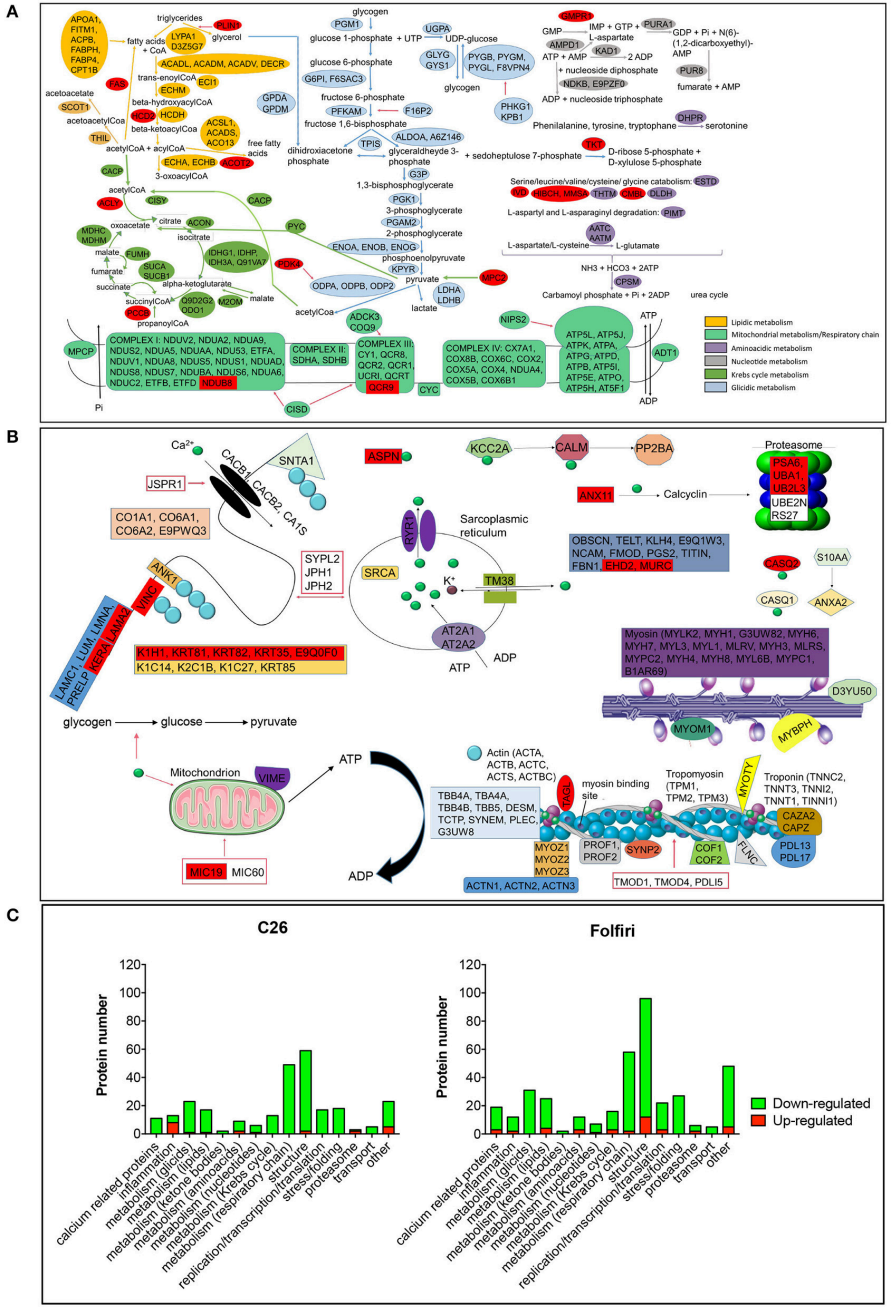
**Major pathways affected in cancer- and drug-induced cachexia**. **(A)** Proteins belonging to any metabolic pathway are indicated and classified as shown in color legend (right). Proteins up-regulated in almost one comparison (C26 vs. control or Folfiri vs. vehicle) are shown in red. All other proteins reported are down-regulated. **(B)** Structural proteins, calcium- and proteasome-associated proteins affected by either cancer or chemotherapy. **(C)** Number of proteins taking part to any of the major pathways affected in cancer- and chemotherapy-induced cachexia. Up-regulated proteins are reported in red, down-regulated proteins are shown in green.

**Figure 4:**
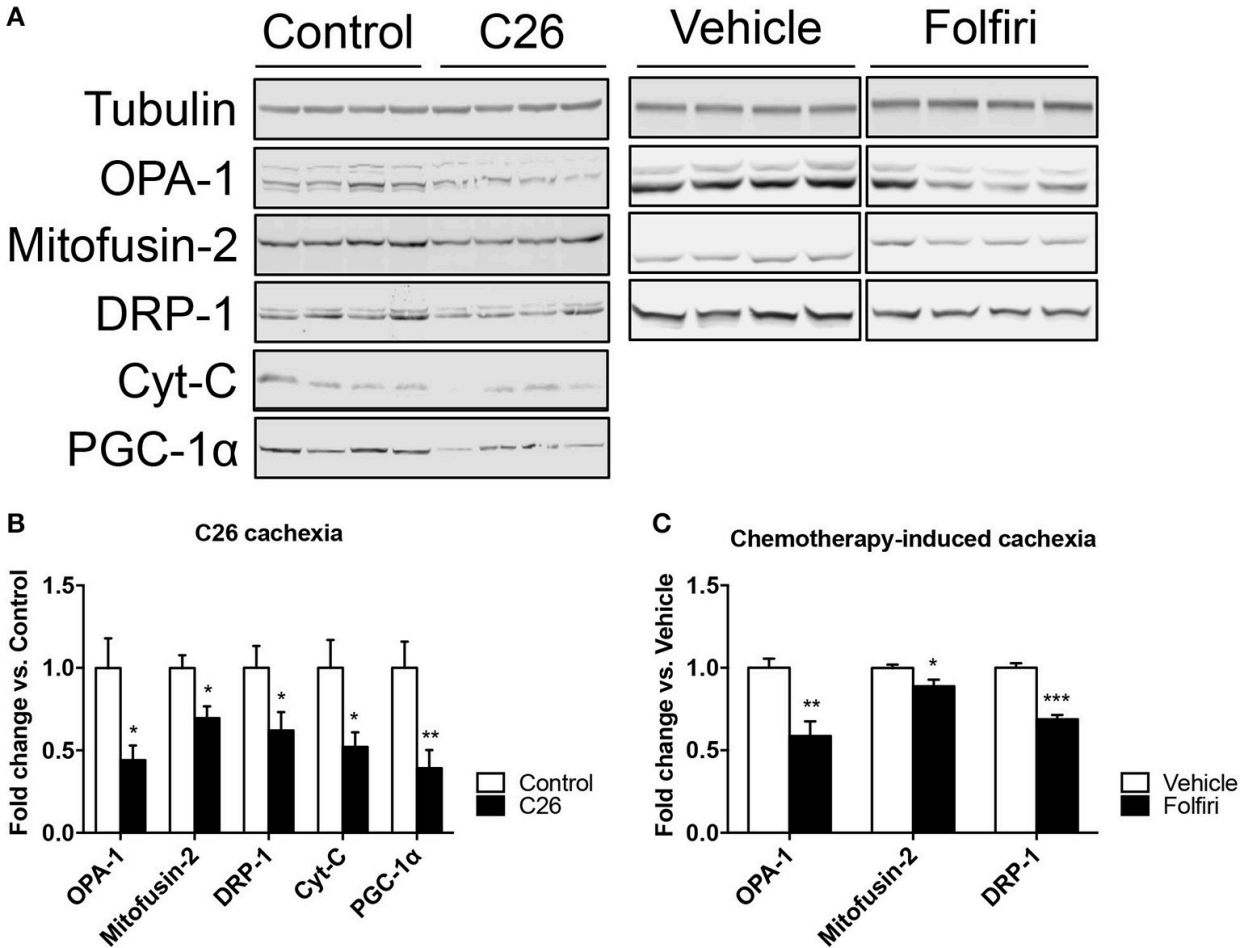
**The expression of markers of mitochondrial fusion, fission and biogenesis is affected by tumor and drug-induced cachexia. (A)** Representative western blotting for OPA-1, Mitofusin-2, DRP-1, Cytochrome-C (Cyt-C), and PGC-1α in the muscle of C26 hosts or mice exposed to Folfiri. **(B,C)** Quantification of the bands (*n* = 4). Significance of the differences: *p < 0.05, **p < 0.01, ***p < 0.001 vs. Control or Vehicle.

The IPA-based analysis performed on the proteins detected in both datasets identified a series of pathways that were similarly affected by both cancer and chemotherapy (Figure 2). In particular, the most represented among the Top-20 pathways influenced by either tumor growth or chemotherapy treatment were associated with mitochondrial dysfunctions, but also alterations of oxidative phosphorylation, TCA cycle, epithelial and tight junction signaling, glycolysis, fatty acid β-oxidation and protein kinase A (Figures 2, 3A; Table S4). Here we show that proteins taking part to the β-oxidation were markedly reduced in the C26-bearing animals, while regulators of the synthesis of fatty acids, such as FAS, TKT and PDK4, were significantly up-regulated (Figures 3A,C; Table S4). Similarly, members of the respiratory chain, such as NDUS6, NDU5, and CISD1, were not detected in the muscle of tumor hosts, suggesting that the energetic metabolism was severely compromised (Figures 3A,C; Table S4). Interestingly, all proteins modulated in the Folfiri dataset were drastically down-regulated with few exceptions, namely several enzymes involved in the metabolism of lipids (PLIN1, HSD17B10, FASN, and ACOT2) or amino acids, such as leucine and valine (IVD, HIBCH, ALDH6A1), the GMP reductase 1 (GMPR) involved in the synthesis and conversion of nucleotides, two regulators of the Krebs cycle (MCP2 and PCCB), and two members of the mitochondrial respiratory chain (NDUFB8 and UQCR10) (Figures 3A,C). In line with these and previous observations (Pin et al., 2015; Barreto et al., 2016), alterations of muscle mitochondrial homeostasis, as suggested by the levels of markers of mitochondrial fusion (OPA-1, mitofusin-2), fission (DRP-1) and biogenesis (Cytochrome-C, PGC-1α), were displayed in the muscle of both C26 hosts and animals exposed to chemotherapy (Figure 4).

In line with previous observations (Costelli and Baccino, 2003; Fearon et al., 2012), we also show that the levels of PSMA6 and UBA1, major proteasomal components, were increased in the muscle of Folfiri-treated animals. Similarly, UBA1 and UB2L3, enzymes associated with the proteasome system, were also up-regulated in the muscle of C26 hosts (Table S4).

Of note, epithelial and tight junction signaling, as well as actin cytoskeleton and calcium signaling, were modulated following either C26 growth or Folfiri treatment (Figures 2, 3B,C; Table S4). In particular, 15 calcium-binding proteins were markedly down-regulated by Folfiri administration, thus suggesting a deregulation of these pathways (Figures 3B,C; Table S4). Interestingly, structural proteins, such as KERA and LAMA2, were up-regulated in the muscle of both Folfiri-treated animals and tumor-bearing mice (Figures 3B,C; Table S4), unlike other proteins, such as MYOZ2, overexpressed in the muscle of C26 hosts and, conversely, down-regulated in the muscle of chemotherapy-treated animals (Table S4). Interestingly, proteins of the 14-3-3 family were down-regulated both in tumor hosts and Folfiri-treated animals (Table S4).

Furthermore, in line with our previous findings (Bonetto et al., 2011), the expression of the majority of the identified positive acute phase response (APR) proteins (CO3, FIBA, FIBB, FIBG, and HPT) was more elevated in the muscle of C26 hosts with respect to the controls, while all negative APRs (TTHY, TRFE and ALBU) were down-regulated (Figure 3C; Table S4). Also, in the presence of chemotherapy, the expression of a number of proteins associated with inflammatory pathways was affected, although the large majority of these mediators were generally down-regulated (Figure 3C; Table S4).

### Similar mechanisms are likely responsible for muscle wasting in both C26- and folfiri-associated cachexia

**Figure 5:**
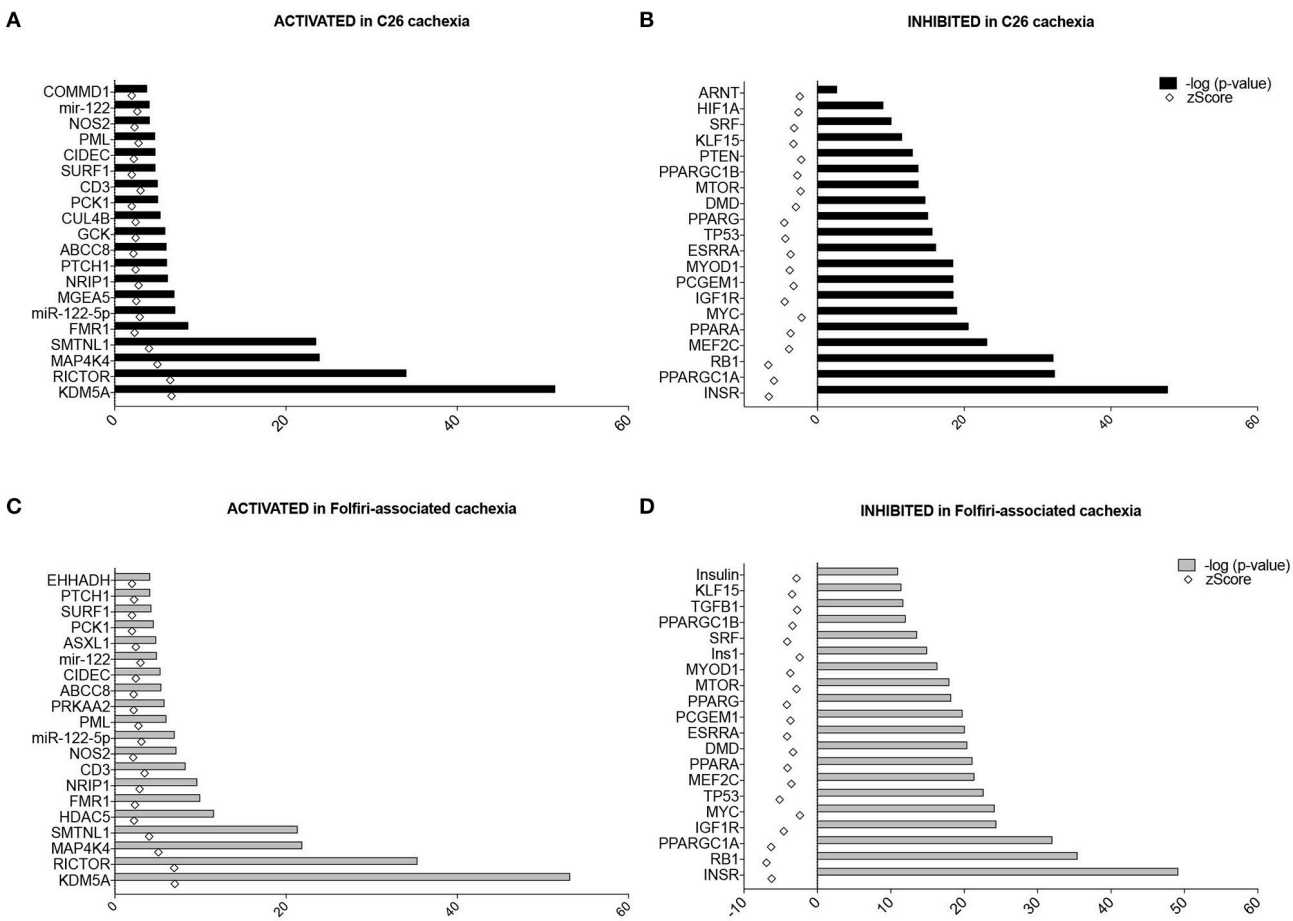
**Upstream regulators in C26- and Folfiri-induced cachexia**. The IPA-mediated analysis identified several upstream regulators ranked based on their overlap *p*-value, whose activation (*z*-score > 2) or inhibition (*z*-score < −2) is associated with the phenotype observed. Top panel: Top-20 upstream regulators activated **(A)** or inhibited **(B)** in C26 cachexia. Bottom panel: Top-20 upstream regulators activated **(C)** or inhibited **(D)** in Folfiri-associated cachexia.

The “Upstream Regulator Analysis” predicted which transcriptional regulators are involved upstream of the changes observed and whether they are likely activated (*z*-score > 2) or inhibited (*z*-score < −2). In particular, among the Top-20 upstream regulators expected to be activated in both datasets, the histone lysine demethylase KDM5A, the mTORC2 subunit RICTOR, the mitogenic-activated protein kinase isoform 4 (MAP4K4), and the contraction regulator Smoothelin-like 1 (SMTNL1) showed the highest *p*-value, providing evidence of a statistically significant overlap between our data and the pathways generally associated with these transcription factors (Figures 5A–C). Conversely, among the Top-20 upstream regulators characterized by a *z*-score < −2 (i.e., likely inhibited) in the skeletal muscle of mice either carrying the C26 tumor or treated with Folfiri, the insulin receptor (INSR), the Peroxisome Proliferator-Activated Receptor Gamma Co-activator 1 Alpha (PPARGC1A, also known as PGC1α) and the tumor suppressor gene RB1 were the highest ranked, whereas the insulin-like growth factor-1 receptor (IGF1R), the regulators of muscle differentiation MYOD1 and MEF2C, as well as other members of the Peroxisome Proliferator-Activated Receptor Gamma family were also identified with lower *p*-values (Figures 5B–D). Of note, no major difference between the two datasets were reported, thus further supporting the idea that similar mechanisms contribute to muscle wasting in both experimental conditions.

### Muscle disorders and alterations of energy production and nucleotide metabolism are associated with cachexia due to cancer or chemotherapy

**Figure 6:**
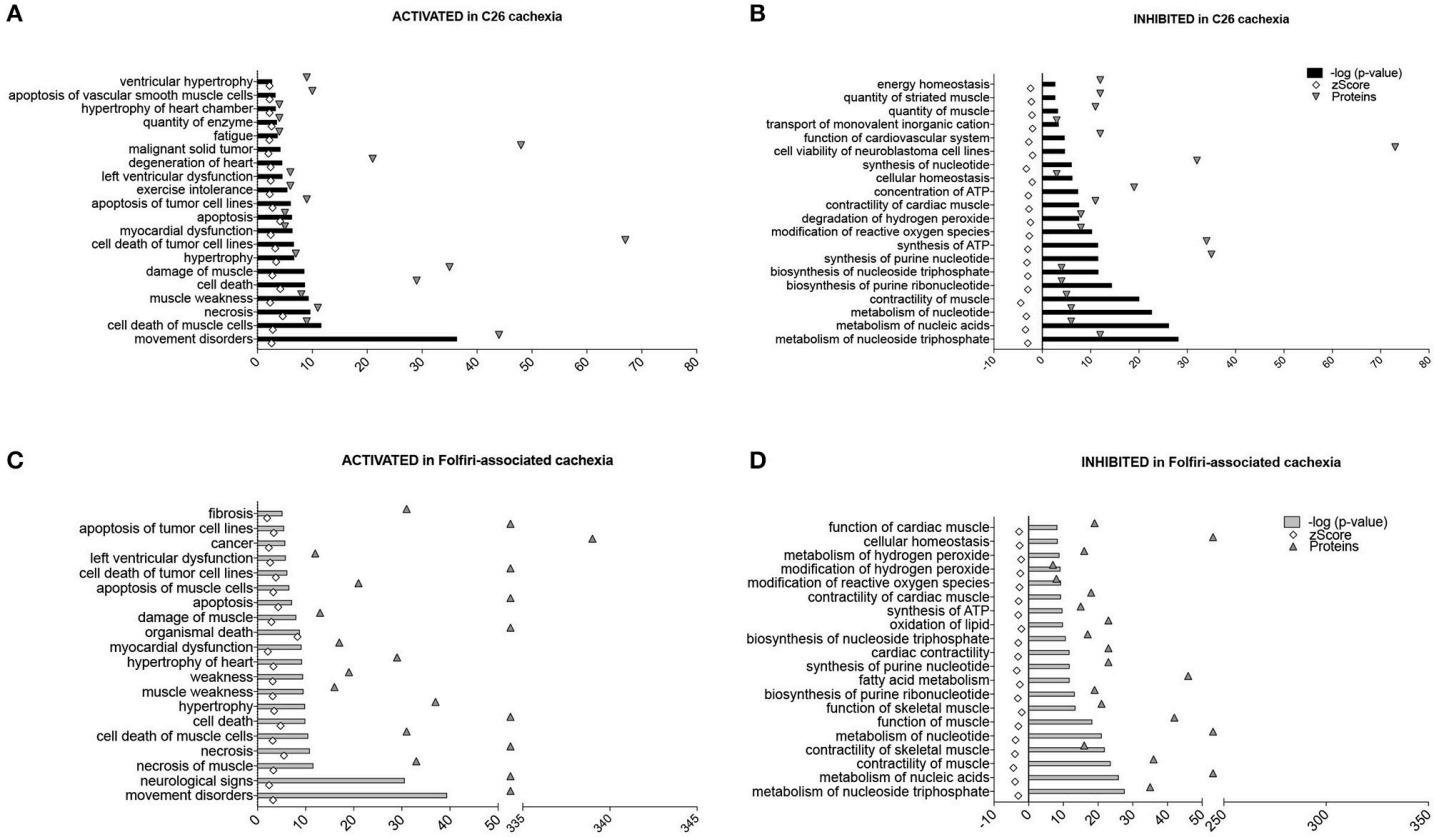
**Diseases and functions associated with C26- and Folfiri-induced cachexia**. The IPA software identified diseases and functions, ranked based on their overlap *p*-value, expected to be activated (*z*-score > 2) or inhibited (*z*-score < −2) in the C26 and Folfiri datasets. Top panel: diseases and functions activated **(A)** or inhibited **(B)** in C26 cachexia. Bottom panel: diseases and functions activated **(C)** or inhibited **(D)** in Folfiri-associated cachexia. Only the Top-20 diseases and functions are reported in the diagram, along with the number of correlated proteins (triangles).

The “Disease and Function Analysis” anticipated which alterations were likely associated with the protein changes reported in the muscle of tumor hosts or in mice exposed to chemotherapy. Interestingly, in both datasets, movement disorders, damage or death of muscle cells and muscle weakness/fatigue were activated (*z*-score > 2), (Figures 6A–C). Further, cardiac dysfunctions seemed to be associated exclusively with tumor growth (Figure 6A), consistent with the decrease in heart mass shown in Table 1. Similarly, the evidence of drug-related neurotoxicity was reported only in the Folfiri dataset (Figure 6B). Conversely, inhibition of nucleotide metabolism and synthesis of ATP, as well as reduced muscle function and modification of ROS, were predicted in both experimental conditions (Figures 6B–D), while alterations of fatty acid metabolism and lipid oxidation were only associated with the chemotherapy treatment (Figure 6D).

## Discussion

Cachexia is a devastating syndrome associated with many disease states, such as cancer, congestive heart failure, diabetes, kidney failure, and HIV/AIDS (Bonetto et al., 2012; Fearon et al., 2012; Dutt et al., 2015). Cancer cachexia is characterized by systemic inflammation, negative protein and energy balance, and an involuntary loss of lean body mass, with or without wasting of adipose tissue (Aoyagi et al., 2015). Muscle weakness has been postulated to occur due to a combination of muscle breakdown, dysfunction, and a decrease in the ability to repair (Isaac et al., 2016). Effective therapies are presently limited, whereas the removal of the primary tumor remains the only definitive treatment strategy. The idea that anticancer treatments may also result in muscle atrophy is currently being debated. Along this line, we recently reported that chemotherapy regimens utilized for the therapy of colorectal cancer, such as Folfox and Folfiri, drive alterations consistent with muscle wasting and muscle weakness (Barreto et al., 2016). Despite this, the mechanisms responsible for muscle loss in the presence of anticancer treatments are not completely known. Furthermore, it is not clear whether similar mechanisms are activated in the presence of either cancer or chemotherapy, thus leading to muscle wasting.

It has been suggested that anorexia, i.e., the reduced or loss of the desire to eat, may represent one of the major causes associated with body and muscle weight loss in oncologic patients (Molfino et al., 2010). Despite the fact that anorexia has been shown to play a role in the development of cachexia in several experimental models, in the present research work we assessed the proteomic profiling in animals only based on the amount of muscle wasting. Moreover, based on available studies, pair-feeding was not performed, particularly because previous results have shown that muscle protein waste is mainly associated with acceleration of protein breakdown rates, regardless of food intake, whereas in pair-fed animals the decrease of skeletal muscle protein content is mainly due to impaired protein synthesis (Tessitore et al., 1993). Therefore, reduced food intake and metabolic competition by the tumor do not seem to justify the hypercatabolic state in the tumor hosts. This view also is shared by the ground-breaking report from Lecker et al. (2004). Similarly, Garcia et al. (2013) showed that cisplatin administration normally affects food intake. However, pair feeding experiments carried out in the same report showed that chronic administration of cisplatin did not induce anorexia and that animals receiving chemotherapy were showing exacerbated body weight loss, regardless of their food intake (Garcia et al., 2013). More recently, in another model we showed that chronic administration of Folfiri to normal animals does not cause anorexia and is only responsible for acute toxicity associated with sudden and temporary drops in food intake, while, on the contrary, the cumulative food intake does not differ from the vehicle-treated animals (Barreto et al., 2016).

In the present experimental work the proteomic analysis performed in skeletal muscle revealed a similar impairment of several metabolic pathways and muscle structures, also consistent with previously published observations (Fontes-Oliveira et al., 2013; Pin et al., 2015; Barreto et al., 2016). Along this line, the large majority of all metabolic enzymes, particularly those associated with the maintenance of the aerobic catabolism (i.e., Krebs cycle and respiratory chain), were drastically down-regulated in both datasets, further supporting the idea that muscle wasting due to chemotherapy administration can be defined as real cachexia. Indeed, oxidative pathways and mitochondrial abnormalities with consequent decreased production of ATP are already well documented features of cachexia and suggest that changes in these pathways might also contribute to muscle weakness, as frequently observed in association with chemotherapy (Fontes-Oliveira et al., 2013; Argilés et al., 2015; Barreto et al., 2016; Carson et al., 2016). In addition, mitochondrial alterations associated with decreased expression of markers of mitochondrial fusion and fission that are normally involved in the maintenance of the integrity and plasticity of the mitochondrial network (Pernas and Scorrano, 2016) were also reported in the muscle of both C26 bearers and mice treated with chemotherapy. This is also consistent with previous reports from our group and others that suggest that cachexia is generally associated with severe alterations of the muscle mitochondria, which may contribute to the occurrence of muscle atrophy, muscle weakness, as well as the transition to more glycolytic muscle fibers (Pin et al., 2015; Barreto et al., 2016). Interestingly, mitochondrial dysfunctions and increased oxidative stress have been shown to play a role in causing disruptions of the neuromuscular junctions, thus possibly explaining the occurrence of muscle weakness and fatigue following cancer development or chemotherapy treatment (Ibebunjo et al., 2013). This is also consistent with our data showing abnormal junction signaling in the muscle of tumor hosts and animals treated with Folfiri.

Notably, our data also show that a few significant differences exist between the two experimental conditions. In particular, all the enzymes of the aerobic metabolism contributing to the Krebs cycle or the respiratory chain are markedly down-regulated in the muscle of tumor hosts, while the same response is not observed following chemotherapy administration, where a considerable number of proteins appear up-regulated. Similarly, pathways associated with the lipidic metabolism were enhanced by the presence of the C26 adenocarcinoma and substantially inhibited following Folfiri administration. In particular, here we showed that tumor growth is associated with decreased activation of the β-oxidation, generally associated with the breakdown of lipids and fatty acids. Interestingly, the synthesis of FAS and TKT, normally associated with the synthesis of fatty acids, were significantly up-regulated. This apparent discrepancy with the phenotype observed in tumor hosts, characterized by severe depletion of fat tissues, may actually result from a survival mechanism that attempts to restore the fat stores, which are essential in a conditions associated with reduced energy metabolism. To further support this point, we also showed that members of the respiratory chain were not detectable in the muscle of tumor hosts, suggesting that the energetic metabolism was impaired.

Our analysis also provides evidence of a concerted down-regulation of structural proteins and calcium-related proteins in the muscle of cachectic mice. In particular, alterations of calcium homeostasis have been reported in clinical and experimental cachexia and other inflammation-driven muscle diseases (Isaac et al., 2016), analogously to the impairment of sarcoplasmic structure (Fontes-Oliveira et al., 2013). A large number of calcium-related proteins and almost all structural proteins that were detected were also shown to be down-regulated in tumor-bearing mice, coherently with the loss of skeletal muscle mass and the occurrence of muscle weakness (Bonetto et al., 2009; Waning et al., 2015). Furthermore, proteins of the 14-3-3 family were also shown to be decreased both in tumor hosts and Folfiri-treated animals. Interestingly, these proteins were recently identified as novel myokines required for maintaining myosin content in skeletal muscle (McLean et al., 2015).

A number of other factors in cancer patients are known to increase the catabolic response, leading to unsustainable levels of fat and muscle mobilization and levels of muscle depletion that cause significant morbidity and mortality (Aoyagi et al., 2015). The up-regulation of proteasomal components observed in association with the occurrence of cachexia is consistent with the well-known activation of skeletal muscle degradative systems, such as the ATP-ubiquitin-dependent one (Bossola et al., 2001; Onesti and Guttridge, 2014). This has also been suggested by the overexpression of muscle-specific ubiquitin ligases, as previously reported in conditions associated with cancer cachexia (Lecker et al., 2004). Conversely, we recently showed that mechanisms other than the ones associated with the activation of proteasome-dependent muscle catabolism are responsible for muscle wasting after Folfiri treatment (Barreto et al., 2016). Regardless, here we show that the levels of major proteasomal components were increased in the muscle of Folfiri-treated animals. Similarly, enzymes associated with the proteasome system were also up-regulated in the muscle of C26 hosts. The discrepancy with our previous data may also suggest that the proteasome-dependent systems might have been involved in promoting chemotherapy-dependent muscle depletion at earlier time points, consistent with findings associated with cachexia (Lecker et al., 2004).

In line with previous reports, a robust skeletal muscle APRs transcriptomic response in association with the activation of muscle catabolism was confirmed in the muscle of C26-bearing mice (Bonetto et al., 2011). Also in this case, the response associated with tumor growth was more coordinated than that following administration of Folfiri. Inflammation and high APR levels are considered a hallmark of cancer cachexia, and an integrated physiological response of substrate mobilization driven by inflammation was proposed as mainly responsible for the development of cachexia (Aoyagi et al., 2015). Despite this, the specific mechanisms by which these cytokines produce skeletal muscle dysfunction remain partially undefined (Isaac et al., 2016). It has been hypothesized that hepatic synthesis of positive acute phase response proteins using amino acids liberated from skeletal muscle proteins is a major driver of skeletal muscle proteolysis (Bonetto et al., 2011). In particular, the levels of fibrinogen expressed in liver vs. muscle in this experimental model suggest that muscle might be a greater source of APR proteins than liver (Bonetto et al., 2011).

In the present work, we show that most of the APR proteins are evenly increased in the muscle of mice carrying a tumor or chronically administered chemotherapy, thus supporting the idea that amino acids freed from skeletal muscle structural proteins through proteolysis would be re-synthesized into these secreted proteins and exported from the cell, possibly contributing to muscle wasting (Bonetto et al., 2011). Altogether, this might suggest that the mechanisms responsible for muscle depletion in the presence of a tumor are also playing a role in promoting muscle wasting upon administration of chemotherapy. In particular, and coherent with our previous findings (Bonetto et al., 2011), a large number of proteins associated with inflammatory pathways was affected both in the presence of cancer or chemotherapy, although in the latter the large majority of these mediators were generally down-regulated.

In conclusion, in the present study we aimed at investigating whether *in vivo* chemotherapy administration could drive the development of cachexia similarly to cancer alone. In particular, in order to unravel the direct modulatory effects of either cancer or chemotherapy on muscle proteome we analyzed the proteomic profiling in the skeletal muscle of C26 tumor hosts or animals exposed to Folfiri. Our study design did not take into consideration the complexity of the interactions between tumor- and chemotherapy-driven mediators, thus apparently representing a limitation. Despite recognizing the importance of future investigations particularly designed to fill this gap of information, we believe this approach was required to assess the effects that are exclusively dependent on the use of anticancer drugs and to definitively include the derangements associated with chemotherapy treatment among the conditions characterized by the occurrence of a cachectic phenotype. Along this line, the data in the present study showed remarkable similarities to the proteomic signatures of cachectic muscles from mice carrying tumors or exposed to chemotherapy, thus further validating the idea that anticancer therapies play a substantial role in causing muscle wasting and muscle weakness, similar to cancer. Analogously, the expected disease pattern associated with the described phenotypes was similar in both experimental conditions, which is consistent with the state of activation of the putative upstream regulators. Of note, signs of neurotoxicity were expected exclusively after Folfiri administration, which is consistent with previous findings that report chemotherapy-related neurotoxicity and muscle weakness (Cordier et al., 2011; Barreto et al., 2016; Taillibert et al., 2016). Ultimately, we showed that dysfunctions of the mitochondrial metabolism represent the main consequence associated with the development of cachexia, thereby corroborating the idea that strategies aimed at protecting the muscle mitochondrial pool may, at the same time, contribute to preserve muscle mass and muscle function in the occurrence of cancer or in association with chemotherapy. Based on our results, future studies will warrant the combination of strategies aimed to both counteract tumor growth and reduce the side effects of chemotherapy.

## Author contributions

Conceived and designed the experiments: RB and AB; Performed the experiments: RB, GM, and AB; Analyzed the data: GM, FW, and AB; Contributed reagents/materials/analysis tools: FW, FN, and TZ; Wrote the paper: GM, FW, and AB.

### Conflict of interest statement

The authors declare that the research was conducted in the absence of any commercial or financial relationships that could be construed as a potential conflict of interest.

## Supplementary material

Table S1 **Detected proteins in the quadriceps muscle of C26-bearing mice**.

Table S2 **Detected proteins in the quadriceps muscle of mice treated with Folfiri**.

Table S3 **Peptide identification and protein coverage**.

Table S4 **Proteomic profiling and pathway analysis of differentially expressed proteins in the skeletal muscle of tumor-bearing mice or mice treated with chemotherapy**.

## Abbreviations

C26: Colon-26 adenocarcinoma
LC-MS/MS: liquid chromatography tandem mass spectrometry
5-FU: 5-fluorouracil
LV: leucovorin
ROS: reactive oxygen species
TCA: tricarboxylic acid
ATP: adenosine triphosphate
MAPK: mitogen-activated protein kinase
APR: acute phase response.
